# Several *Plasmodium vivax* relapses after correct primaquine treatment in a patient with impaired cytochrome P450 2D6 function

**DOI:** 10.1186/s12936-020-03326-1

**Published:** 2020-07-17

**Authors:** Alexandra Martin Ramírez, Carlos Lombardia González, Tamara Soler Maniega, Ángela Gutierrez Liarte, Diego Domingo García, Marta Lanza Suárez, María Josefa Bernal Fernández, José Miguel Rubio

**Affiliations:** 1 Malaria and Parasitic Diseases Laboratory, National Microbiology Center, Instituto de Salud Carlos III, Madrid, Spain; 2 Genetics Department, CatLab, Barcelona, Spain; 3 Microbiology and Parasitology Department, Hospital Universitario de la Princesa, Madrid, Spain; 4 Internal Medicine Department, Hospital Universitario de la Princesa, Madrid, Spain

**Keywords:** *Plasmodium vivax*, Malaria, Relapse, CYP2D6, Primaquine

## Abstract

**Background:**

*Plasmodium vivax* malaria is characterized by the presence of dormant liver-stage parasites, called hypnozoites, which can cause malaria relapses after an initial attack. Primaquine, which targets liver hypnozoites, must be used in combination with a schizonticidal agent to get the radical cure. However, relapses can sometimes occur in spite of correct treatment, due to different factors such as a diminished metabolization of primaquine.

**Case presentation:**

In January 2019, a 21 years old woman with residence in Madrid, returning from a trip to Venezuela with clinical symptoms compatible with malaria infection, was diagnosed with vivax malaria. Chloroquine for 3 days plus primaquine for 14 days was the elected treatment. Two months later and after a second trip to Venezuela, the patient presented a second *P. vivax* infection, which was treated as the previous one. A third *P. vivax* malaria episode was diagnosed 2 months later, after returning from a trip to Morocco, receiving chloroquine for 3 days but increasing to 28 days the primaquine regimen, and with no more relapses after 6 months of follow up. The genotyping of *P. vivax* in the three malaria episodes revealed that the same strain was present in the different relapses. Upon confirmation of correct adherence to the treatment, non-description of resistance in the infection area and the highly unlikely re-infection on subsequent trips or stays in Spain, a possible metabolic failure was considered. *CYP2D6* encodes the human cytochrome P450 isoenzyme 2D6 (CYP2D6), responsible for primaquine activation. The patient was found to have a CYP2D6*4/*1 genotype, which turns out in an intermediate metabolizer phenotype, which has been related to *P. vivax* relapses.

**Conclusions:**

The impairment in CYP2D6 enzyme could be the most likely cause of *P. vivax* relapses in this patient. This highlights the importance of considering the analysis of CYP2D6 gene polymorphisms in cases of *P. vivax* relapses after a correct treatment and, especially, it should be considered in any study of dosage and duration of primaquine treatment.

## Background

The major human malaria parasites are *Plasmodium falciparum* and *Plasmodium vivax*. According to the World Health Organization (WHO), about 3.3% of malaria cases in 2018 were caused by *P. vivax* [1]. This is highly prevalent in Southeast Asia and South America, representing about 75% of malaria cases in America, 50% of cases in Southeast Asia and 29% in Eastern Mediterranean Region [1].

Spain was a malaria-endemic country until 1964, when malaria elimination was declared [2]. Since then, most malaria cases are imported. However, there have been two cases of autochthonous vivax malaria: one in a 48 years old woman in 2010 [3] and one in a 62 years old man in 2014 [4].

Unlike *P. falciparum, P. vivax* exhibits dormant liver-stage parasites (hypnozoites), which are responsible of malaria relapses weeks or months after the initial attack [5]. In the past, vivax malaria was considered as the benign tertian malaria, with mild symptoms, such as fever, chills or headache; in contrast with the malignant tertian malaria identity attached to the falciparum malaria. However, more and more studies point out *P. vivax* as a cause of severe malaria, associated with potentially like-threatening conditions [6, 7], and most acute cases of *P. vivax* are originated from hypnozoites rather than sporozoites [8]. Radical treatment targeting not only the blood stages of the parasite, but also the dormant liver-stage parasites is essential to prevent future relapses. The recommended treatment of uncomplicated vivax malaria is chloroquine, in areas with chloroquine-susceptible infections; or artemisinin-based combination therapy (ACT) in areas with chloroquine-resistant infections [9]. The drug of choice against hypnozoites is primaquine, an 8-aminoquinoline anti-malarial agent [5], although recently, tafenoquine has shown efficacy for the radical cure of vivax malaria [10].

The common use of chloroquine for treating *P. vivax* infection led to the appearance of resistance years ago. The first suggestion of *P. vivax* resistance to chloroquine was reported in Papua New Guinea in 1989 [11, 12] and since then, resistance has spread to other countries [13]. Nevertheless, chloroquine continues as the first-line treatment for *P. vivax* in the Americas, in the Eastern Mediterranean Region and in most countries of Southeast Asia, where still continues to demonstrate high efficacy, except for Indonesia, Myanmar and Timor-Leste [1].

While on the contrary, primaquine has remained since its introduction in 1952 as anti-malaria treatment [14], and no real evidence of primaquine resistance has been identified at the moment. Some cases of primaquine resistance, especially in Southeast Asia, have been described. The first one was in the Chesson strain of *P. vivax* [15]; however, it was not possible to exclude other factors for the relapses [16]. In addition, different factors have been indicated as responsible of primaquine failures. The proper dose has been pointed out as a key in decreasing the risk of relapsing. The absence or very low doses of primaquine have been associated with recurrences [17, 18], but some studies have shown that the standard adult dose of primaquine (15 mg/day for 14 days) has not been effective and higher doses of primaquine might be needed for a complete parasite eradication [19]. In addition to using the proper dose of primaquine, another important factor is the adherence to medication regimen. A previous study reported more relapses in patients who missed primaquine doses compared to those who completed the primaquine 14-day course, in *P. vivax*-infected individuals [20]. Another factor to take into account is the possibility of failures in the metabolic activation of primaquine. To eliminate *Plasmodium* hypnozoites, primaquine has to be activated by the isoenzyme 2D6 of human cytochrome P450 (CYP2D6), which is converted to oxidized metabolites which are responsible for the anti-hypnozoites activity [21–23]. CYP2D6 is responsible for the metabolism of 20–25% of clinically used drugs, and there are more than 46 known major *CYP2D6* alleles which can be determined and predict a CYP2D6 phenotype of metabolization (poor, intermediate, normal and ultrarapid) [22]. It has been suggested that a reduced primaquine metabolism due to impairments in CYP2D6 function may be related to *P. vivax* relapses [24–27].

The aim of this study is to elucidate the reason for several recurrences of vivax malaria in a patient after a correct primaquine treatment, including the possible role of the specific allelic combination of the human CYP2D6.

## Case presentation

In January 2019, a 21 years old woman of Venezuelan origin with residence in Madrid, Spain, without any previous history of striking disease, attended the *Hospital Universitario de la Princesa* in Madrid with episodes of fever, headache, chills and cough for 1 week and diarrhoea for the previous 2 days. She referred to a trip to a coastal area in Venezuela (Higuerote) the previous month, coming back 10 days before she attended to the hospital. *Plasmodium vivax* malaria was diagnosed after blood thin smear and rapid diagnostic test (RDT) (BinaxNOW Malaria, Abbott) in the Microbiology and Parasitology Department of the hospital. The sample was sent to the Malaria and Parasitic Diseases Laboratory (MAPELab) of *Instituto de Salud Carlos III*, for further confirmation by molecular methods. Molecular species identification was performed by a nested multiplex PCR (NM-PCR) assay targeting the small sub-unit rDNA gene (SSU rDNA), which comprises two PCR steps: a first PCR assay identifying *Plasmodium* spp. and human DNA as the internal control; and a second PCR assay targeting *P. falciparum, P. vivax, Plasmodium ovale, Plasmodium malariae* [28] and *Plasmodium knowlesi* [29]. The NM-PCR confirmed a *P. vivax* infection.

The patient was firstly treated with oral chloroquine for 3 days and with 14 days of primaquine phosphate (Durbin LTD, United Kingdom) at the WHO recommended dosage (0.25 mg/kg/day) [9], after checking the normal glucose-6-phosphate dehydrogenase function. No other treatments were prescribed. The patient recovered totally and in the follow-up 1 month later she was asymptomatic and without anaemia or other analytical alterations. Two months after the first episode, the patient came back to the hospital with fever, headache and chills after returning from a trip of 12 days to Caracas (Venezuela). Microscopy, RDT and NM-PCR confirmed a *P. vivax* infection again. The prescribed treatment was the same to the previous episode, chloroquine plus primaquine for 14 days. The patient progressed favourably and the blood smear and the immunochromatography for malaria were negative 1 week later. In the follow-up 1 month later the malaria conventional diagnosis was negative again. However, 2 months later, and after a trip to Morocco, the patient returned to the hospital with fever, chills, fatigue and nausea. Once again, thin blood smear, RDT and NM-PCR were positive for *P. vivax* infection. Given this third consecutive malaria infection, and due to a high possibility of a therapeutic failure, the treatment was modified to 3 days of chloroquine with 28 days of primaquine. The clinical and parasitological follow-up 1 week and 1 month later were negative for the malaria diagnostic tests (microscopy and RDT). Six months later (February 2020) the patient was asymptomatic and the microscopy and RDT for malaria were negative.

In order to determine if the last two infections were due to recurrences or to re-infections, the *P. vivax* strains of the three malaria episodes of the patient were genotyped. The genotyping was carried out by analysis of three polymorphic regions (F1, F2 and F3) of the *merozoite surface protein*-1 gene of *P. vivax (Pvmsp*-1), by PCR and sequencing. The characterization of the amplified PCR products was performed by analysis of the fragment size, for the polymorphic region F3; and by comparison of the sequences, for polymorphic regions F1 and F2 of *Pvmsp*-1 gene [30].

**Fig. 1:**
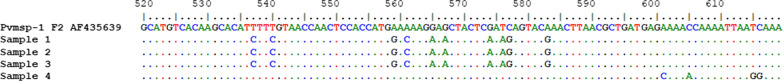
Partial alignment of the patient’s samples sequences (samples 1, 2 and 3) obtained in the *Pvmsp*-1 PCR F2 with a *Plasmodium vivax* reference sequence (accession number AF435639) and another *Plasmodium vivax* strain (sample 4). The alignment is in forward sense (5′–3′). Coloured dots represent an identical nucleotide

**Fig. 2:**
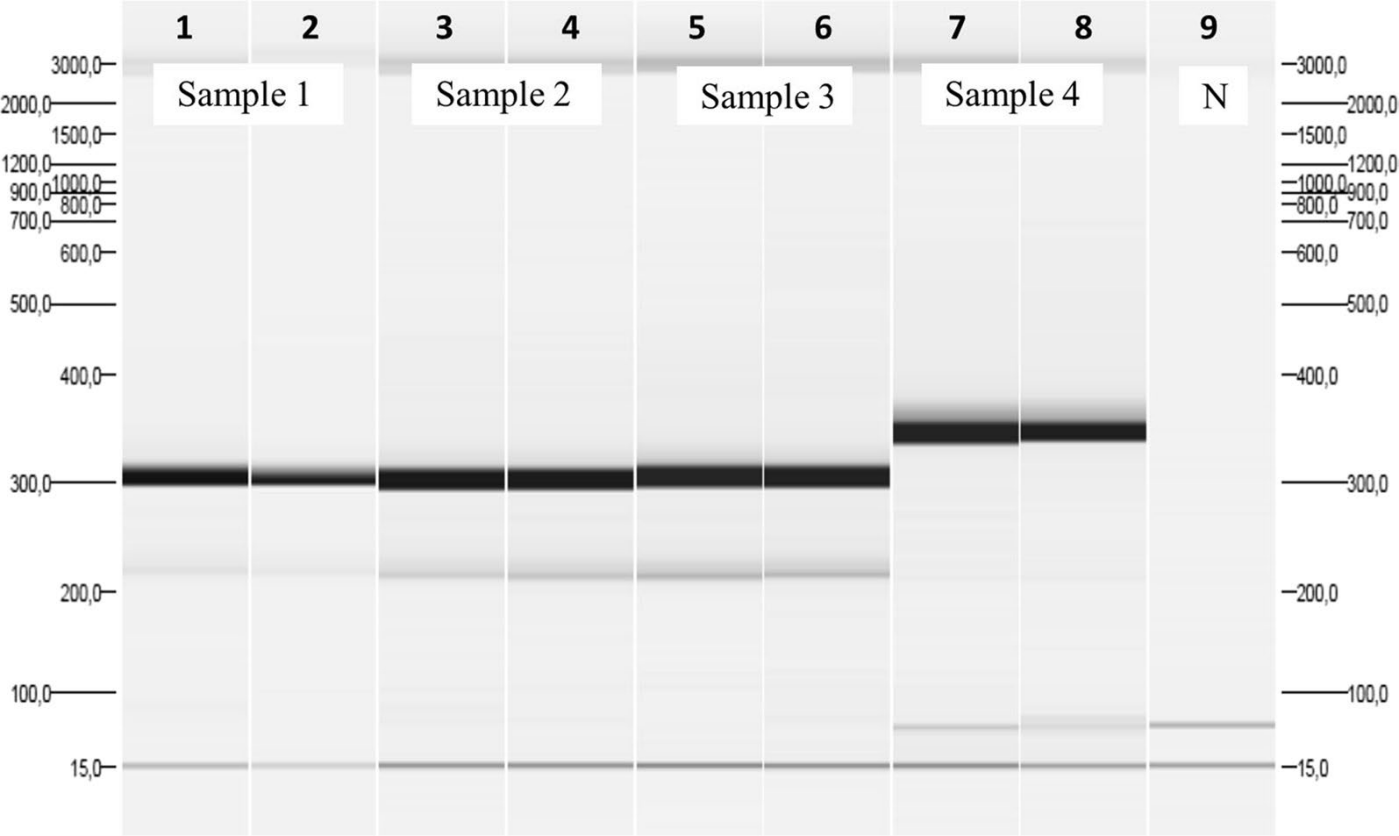
Automatic electrophoresis (QIAxcel, QIAGEN^®^) of the amplified fragments of the region F3 of the *Pvmsp*-1. Samples 1, 2 and 3 belonged to the studied patient. Sample 4 was a *P. vivax* of different origin. Samples from 1 to 4 were amplified by duplicate. `N´ was a negative sample. Fragments of 15 bp and 3000 bp are the internal markers of the automatic electrophoresis

The sequences, obtained from amplification of polymorphic F1 and F2 regions, were aligned using ClustalW multiple alignment of the Bioedit Sequence Alignment Editor, version 7.2.5 [31]. They were compared with sequences of the same polymorphic areas of the *Pvmsp*-1 gene, extracted from GeneBank data base using the AF435639 accession number as the reference sequence. The three sequences corresponding to the patient were equal between themselves and different to most of the compared sequences (Fig. 1). The analysis of PCR product sizes of the *Pvmsp*-1 PCR F3 showed that the patient’s three samples had the same size, meanwhile *P. vivax* control strains showed different sizes (Fig. 2).

The results obtained support that the three malarial episodes were due to recurrences of an initial infection, as the three *P. vivax* strains isolated from the patient were genetically homologous or closely related, and not due to new infections acquired in her successive trips or in Spain. A possible cause of a recurrence is poor adherence to treatment. However, in this case, the patient ensured that she followed the treatment correctly and there were no reasons to think otherwise. Then, the appearance of recurrences, in the context of a correct treatment, led to analysis to establish if the therapeutic failure could be connected to impairment in the metabolism of the primaquine. In order to analyse the genotype of the *CYP2D6* gene of the patient real-time PCR was performed. This technique is able to detect the single nucleotide variants (SNVs) defining the alleles *3 (rs35742686), *4 (rs3892097 and rs1065852), *6 (rs5030655), *9 (rs5030656), *10 (rs1065852), *17 (rs28371706), and *41 (rs28371725) of *CYP2D6*, using TaqMan^®^ probes which detects the specific and wild-type alleles (TaqMan Genotyping Assay, ThermoFisher Scientific); as well as copy number variants (CNVs) and hybrid genes, using specific probes for intron 2 and exon 9 (TaqMan Copy Number Assay, ThermoFisher Scientific). Testing was performed following manufacturer’s instructions. The detection of *5 allele, a large gene deletion, was performed using conventional PCR [32], followed by automatic electrophoresis analysis (DNA High Resolution Gel Cartridge, QIAxcel, QIAGEN^®^). The results showed heterozygous positivity for allele *4 (rs3892097 and rs1065852 were detected). Neither other SNVs nor structural variant of *CYP2D6* were found.

## Discussion

Recurrences of *P. vivax* parasites in peripheral blood may derive from different sources: from sporozoites of a new infection (re-infection), from sub-patent asexual parasitaemia (recrudescence) due to resistance to the treatment or from hepatic hypnozoites (relapse).

The patient could have been re-infected with a new *P. vivax* strain during her subsequent trips to Venezuela and to Morocco, or during her stay in Spain. Venezuela is a malaria-endemic country, with 80% of malaria cases caused by *P. vivax* and with a large increase of malaria cases following the political and economic crisis [1, 33].

On the contrary, Morocco was certified as malaria-free by the WHO in 2010 [34]. Nevertheless, Morocco reports about 100 of imported malaria cases, mostly *P. falciparum* cases originating from sub-Saharan Africa, and still present is the major vector, *Anopheles labranchiae*. However, the probability that the re-infection had occurred during her trip to Morocco is very low, because vectors are present in formerly malarious areas and imported malaria cases are often diagnosed in big cities, far from the areas where the entomological risk is high [35]. On the other hand, Spain is a malaria-free country since the early 1960s [2], with two autochthonous cases in the last 10 years in rural areas in the north of the country [3, 4]. In large cities such as Madrid, where the patient resides, the presence of vectors has not been reported, and therefore the probability of local infection is really negligible.

To elucidate the origin of the *P. vivax* infection after the first episode of malaria, the most common method is genotyping the different strains by PCR and sequencing involving polymorphic regions of specific genes of the parasite [8]; although methods comparing the whole genome sequencing data are more reliable and less susceptible to errors [36]. These molecular genotyping methods are able to discriminate between genetically homologous and heterologous infections, but they are not able to differentiate between a recrudescence or a relapse with a homologous strain [12]. Contrary to general expectation, relapses caused by parasites genetically distinct from those that caused the acute infection have been reported in areas where malaria transmission was low or absent [37]. The genotyping of the three polymorphic regions of the *Pvmsp*-1 gene showed a 100% concordance in fragment size and sequence in the three clinical episodes. This supports that the patient acquired the initial infection in her first trip to Venezuela and the successive episodes were due to recrudescences or relapses.

Recrudescence due to a chloroquine failure, which means the re-appearance of parasitaemia from the residual blood stage parasites following the therapy, was another possibility for the second and third malaria episodes. However, this is highly unlikely for several reasons. Firstly, after the first and second malaria episodes there was a clinical cure, with no clinical symptoms; and a parasitological cure, with negative results in microscopy and RDT tests in the follow-ups 1 week and 1 month after starting the treatment. A useful measure of chloroquine resistance is the assessment of parasitaemia by day 28 [12, 13, 38], since no recurrent parasitaemia should be noted by that day in patients taking a complete treatment course with an adequate absorption [12, 39], and recurrences beyond day 28 after the full compliance with standard chloroquine therapy could be relapses by chloroquine-sensitive *P. vivax* [39]. In addition, clearance of parasitaemia assessed by microscopy by day 3 was 100% predictive of chloroquine sensitivity in a previous meta-analysis study [12]. In this case, the patient experienced the recurrence of the infection around two months after the treatment. Secondly, chloroquine resistance was first described in Papua New Guinea and then spread to other parts of the world, including South America [13, 40]. Although Venezuela, the country where the patient probably acquired the infection, has no reported cases of chloroquine resistance at the moment; other nearby countries, as Colombia, Brazil and Guyana, do have reported resistance cases [12], so it is important to be aware and consider this possibility.

Therefore, the most likely explanation is that the cause of the successive recurrences was due to relapses of the first infection. Furthermore, the time from primary attack to first and second relapse was about 2 months, which is compatible with the mean time to relapse established for South America [41].

The relapses may be due to different factors: a lack of adherence to treatment, the use of inappropriate doses, the presence of resistant strains or a failure in treatment related to drug metabolization. The most common cause of a recurrence is poor adherence to treatment. In the case of primaquine, this is highly variable in endemic areas, ranging from 62 to 95% of adherence [42–44]. Nevertheless, the patient of this study reinforced that she had completed the full dosage of primaquine in all episodes, and she attended to all follow-up doctor’s appointments. The use of inappropriate doses also can be a possibility, the absence or very low doses of primaquine have been associated with recurrences [17, 18]. However, the patient received primaquine at a dose of 15 mg/day for 14 days, after the first two infections, which is the preventive treatment recommended by the WHO for *P. vivax* to avoid relapse [9].

On the other hand, some cases of primaquine resistance have been described, but it has been impossible to exclude other causes, as the primaquine dose, the duration of the treatment or the concurrent blood schizonticidal agent administered [14]. In addition, thanks to more recent studies, relapses have been related to impairments in primaquine metabolization.

To eliminate the hypnozoites, primaquine has to be activated by the hepatic isoenzyme cytochrome P450 2D6 (CYP2D6), which is also involved in the metabolism and bioactivation of many other different used drugs [45] and is encoded by a highly polymorphic gene [22]. The human *CYP2D6* genotype can be determined by molecular techniques and, depending on which combination of alleles is present, phenotypes can be predicted. Although there were discordances in translating the *CYP2D6* genotype to a metabolizer phenotype across laboratories, it has been recently standardized [46]. The assigned phenotype is based on an activity score (AS), where each allele has an ‘activity value’ ranging from 0 to 1 (0 for no function, 0.5 for a decreased function, and 1 for a normal function). Poor metabolizer (PM) phenotypes are defined by AS of 0, intermediate metabolizers (IM) are defined by AS from 0 to 1.25, normal metabolizers (NM) are defined by AS from 1.25 to 2.25, and ultrarapid metabolizers (UM) are defined by AS over 2.25 [38]. Recent studies, have established the importance of CYP2D6 polymorphisms on primaquine efficacy [24–27, 47] showing associations between low-activity CYP2D6 phenotypes (poor and intermediate metabolizers) *and P. vivax* relapses. In the study of Bennett et al. [24] two out of 25 patients relapsed after an initial *P. vivax* attack with correct chloroquine and primaquine treatment. These two patients presented intermediate and poor metabolizers of CYP2D6 enzyme, respectively. In addition, a subsequent nested case–control study showed a significant association between the impaired CYP2D6 activity enzyme and the risk of primaquine therapeutic failure in *P. vivax* infected patients [25].

The molecular analysis of *CYP2D6* gene of the patient of this study showed heterozygosity for allele *4, categorized as a non-functional allele [48, 49]. CYP2D6*4 was present in all the Venezuelan populations tested in previous studies [50, 51], and it has similar allele frequencies in populations of Spain and other Latin American countries, as a result of the process of conquest [50]. Other SNVs or structural variant were not detected. However, allele *1, characterized by the absence of any sequence variations, is assigned by default when other SNPs are not detected during testing [52]. Then, the patient presented a CYP2D6 *1/*4 genotype. As *1 allele is a normal function allele (with an activity value of 1), the CYP2D6 *1/*4 genotype turns out in an intermediate metabolizer phenotype (AS = 1) [46]. Therefore, this shows that the patient of this study has an impaired function of CYP2D6 metabolism, resulting in levels of metabolized primaquine inadequate to the task of killing hypnozoites, as prior studies have reported [47, 48]. This fact could also be due to co-morbidities or concomitant medicines that inhibit the cytochrome action, however, our patient did not show other diseases and did not received other treatments, excluding other possible causes.

In this patient, after two episodes of relapse, primaquine regimen was extended from 14 to 28 days, trying to avoid future relapses with a higher dose primaquine regimen, which has been mentioned to be more effective in preventing relapses of *P. vivax* infection [8, 19, 53]. At the moment, the patient has not suffered any more relapses and in the follow-up six months later since the last *P. vivax* episode the patient was asymptomatic and the microscopy and RDT for malaria were negative.

## Conclusion

The results point out that the two recurrences that the patient suffered were more probably due to two relapses from the first infection acquired in Venezuela. Re-infection from different *P. vivax* strains and recrudescence due to chloroquine resistance were discharged by genotyping and clinical and parasitological analysis, respectively. Therefore, the most feasible explanation for relapses is a primaquine therapeutic failure. Low primaquine doses and lack of adherence were ruled out, as well as a possible primaquine resistance due to the low credibility of the described cases and, in addition, due to the origin of the infection. Primaquine has to be activated by the hepatic isoenzyme cytochrome P450 2D6 (CYP2D6), which is highly polymorphic and depending on the alleles has different levels of activity for drug metabolization. The patient showed an impaired function in CYP2D6 enzyme, due to an intermediate metabolizer phenotype related to specific polymorphisms of *CYP2D6* gene, which results in a diminished metabolism. This case report, along with previous studies, points out CYP2D6 as a possible important determinant of efficacy of primaquine against relapse. This highlights the importance of considering the analysis of *CYP2D6* gene polymorphisms in cases of *P. vivax* relapses after a correct treatment and, especially, it should be considered in any study of dosage and duration of primaquine treatment. However, stronger evidence is needed to verify this relationship and to consider other treatment alternatives in cases of *P. vivax* treatment failures.

## Data Availability

Not applicable

## Abbreviations

AS: Activity Score
Bp: Base pair
CNVs: Copy number variants
CYP2D6: Isoenzyme 2D6 of cytochrome P450
CYP2D6: Gene encoding CYP2D6 enzyme
IM: Intermediate metabolizer
MAPELab: Malaria and Parasitic Diseases Laboratory
NM-PCR: Nested multiplex PCR for malaria
NM: Normal metabolizer
PM: Poor metabolizer
RDT: Rapid Diagnostic Test
SNPs: Single Nucleotide Polymorphisms
SNVs: Single nucleotide variants
SSU rDNA: Small subunit of the ribosomal DNA
UM: Ultrarapid metabolizer
